# A Wireless Battery-Free Implant With Optical Telemetry for In Vivo Cortical Stimulation

**DOI:** 10.1109/lsens.2024.3387370

**Published:** 2024-04-12

**Authors:** Abed Benbuk, Diogo Moniz-Garcia, Daniel Gulick, Alfredo Quinones-Hinojosa, Jennifer Blain Christen

**Affiliations:** 1 Department of Electrical, Computer, Energy Engineering, Arizona State University, Tempe AZ 85287 USA; 2 Department of Neurologic Surgery, Mayo Clinic, Jacksonville, FL 32224 USA

**Keywords:** Sensor applications, battery-free, cortical stimulation, dura substitute, implant, optical telemetry, wireless, wireless power transfer (WPT)

## Abstract

We present a 100 *μ*m-thick, wireless, and battery-free implant for brain stimulation through a U.S. Food and Drug Administration-approved collagen dura substitute without contact with the brain’s surface, while providing visible-light spectrum telemetry to track the onset of stimulation. The device is fabricated on a 16 × 6.67 mm^2^ biocompatible parylene/PDMS substrate and is encapsulated with a 2 *μ*m-thick transparent parylene layer that enables the relay of the LED brightness. The *in vivo* rodent testing confirmed the implant’s ability to trigger motor response while generating observable brightness through the skin. The results reveal the prospect of wireless stimulation with enhanced safety by eliminating contact between the implant and the brain, with optical telemetry for facilitated tracking.

## Graphical Abstract

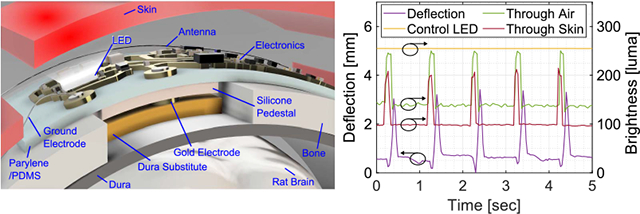

## I. INTRODUCTION

**Fig. 1.**
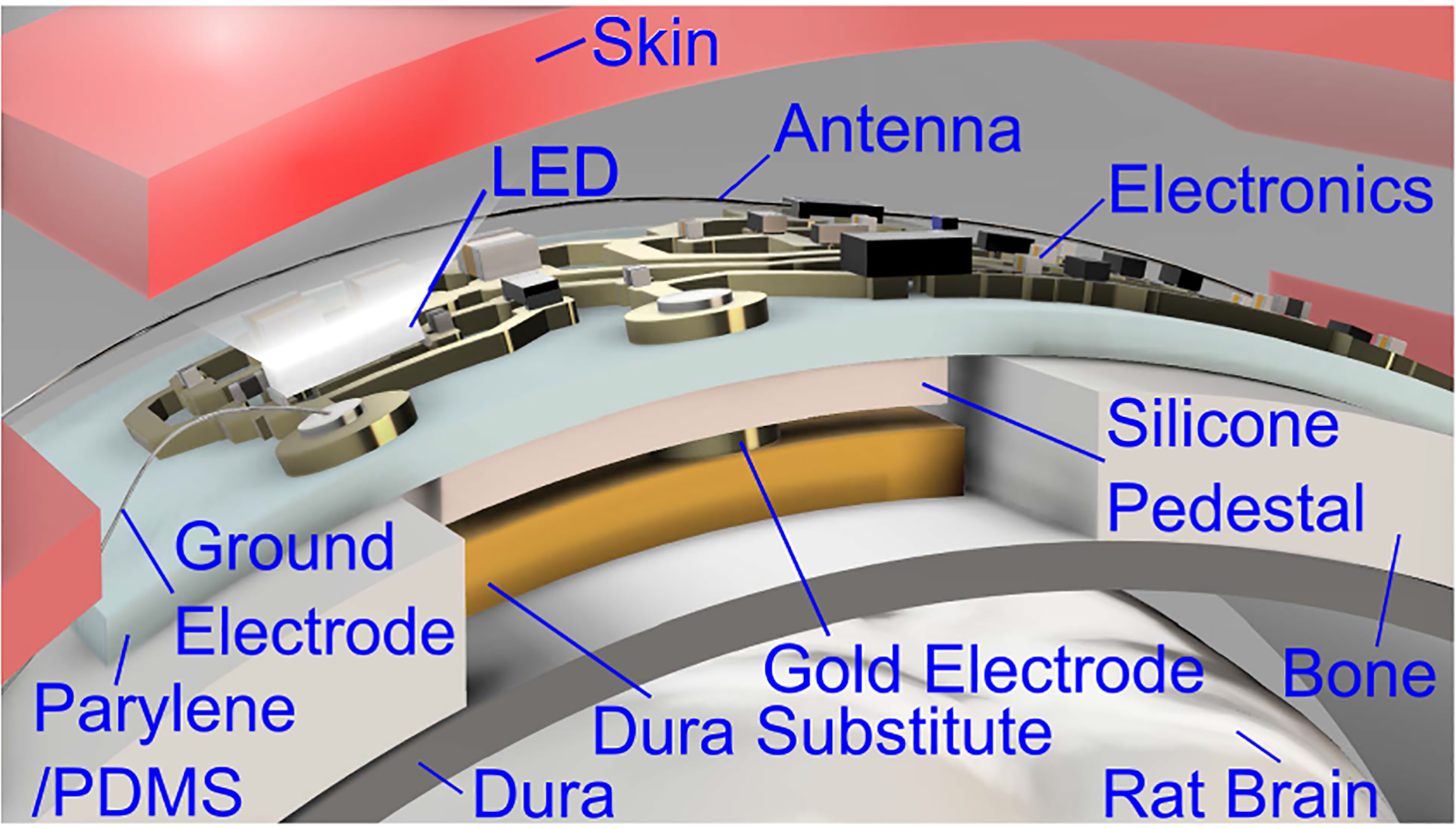
Implant placement for wireless stimulation.

Neural implants with electrical stimulation have great potential and are currently used to treat several conditions, such as movement disorders using deep brain stimulation [1], vagus nerve stimulation [2], as well as emerging uses in psychiatric disorders ranging from depression to addiction [3]. Further, there are exciting results in the brain cancer field suggesting a potential role for implantable devices in the near future [4]. However, significant challenges remain when implementing neural implants, such as the risk of wire movement or breakage, and the need for battery replacement surgeries [5], [6]. Therefore, there is a need to develop wireless, thin, and flexible implants to avoid these complications. Wireless implants rely on antennas [7] or coils [8] to supply power to the implant. Electrical stimulation is delivered via monophasic or biphasic voltage pulses [9], [10], using active electronics, such as microcontrollers, to determine the stimulation parameters [11], [12], or custom integrated circuitry [8]. On the other hand, optogenetics has emerged as an alternative method to deliver stimulation using light-emitting diodes (LED) [13]. Long-term testing requires telemetry to track the onset of stimulation and verify the operation of the implanted device, typically through the skin. Telemetry has been realized at various wavelengths, including radio frequency (RF) [14], visible [11], and infrared (IR) [15], [16], and by other modalities including ultrasound [17]. In these studies, the telemetry unit is powered and operated using a microcontroller or custom integrated electronics. This approach suffers from increased active power consumption and peak thickness of the implant due to the large size of the active electronics, which complicates testing in small anatomy, such as a rodent’s cortex. We developed a wireless, battery-free, and passive (no continuous voltage drain drain, VDD) implant with optical telemetry for in vivo brain stimulation in rodents. The implant can be controlled externally to generate a clinically-relevant stimulation protocol that is composed of bursts of monophasic voltage pulses. The stimulation burst simultaneously powers an LED operating at a wavelength of 477 nm to enable naked-eye verification of device operation and long-term monitoring in rodents. The device is tested in vivo by delivering stimulation to a rodent’s motor cortex, with the positive electrode making contact to a Food and Drug Administration-approved dura substitute material in the craniotomy site above the motor cortex as shown in Fig. 1. The results show that wireless stimulation of the motor cortex through the dura substitute triggers motor response while relaying optical telemetry through the skin. These results enable researchers to administer electronics to combine the therapeutic benefits of the dura substitute with electrical stimulation and optical telemetry for long-term studies in rodents.

## II. MATERIALS AND METHODS

### A. Wireless Battery-Free Implant

**Fig. 2.**
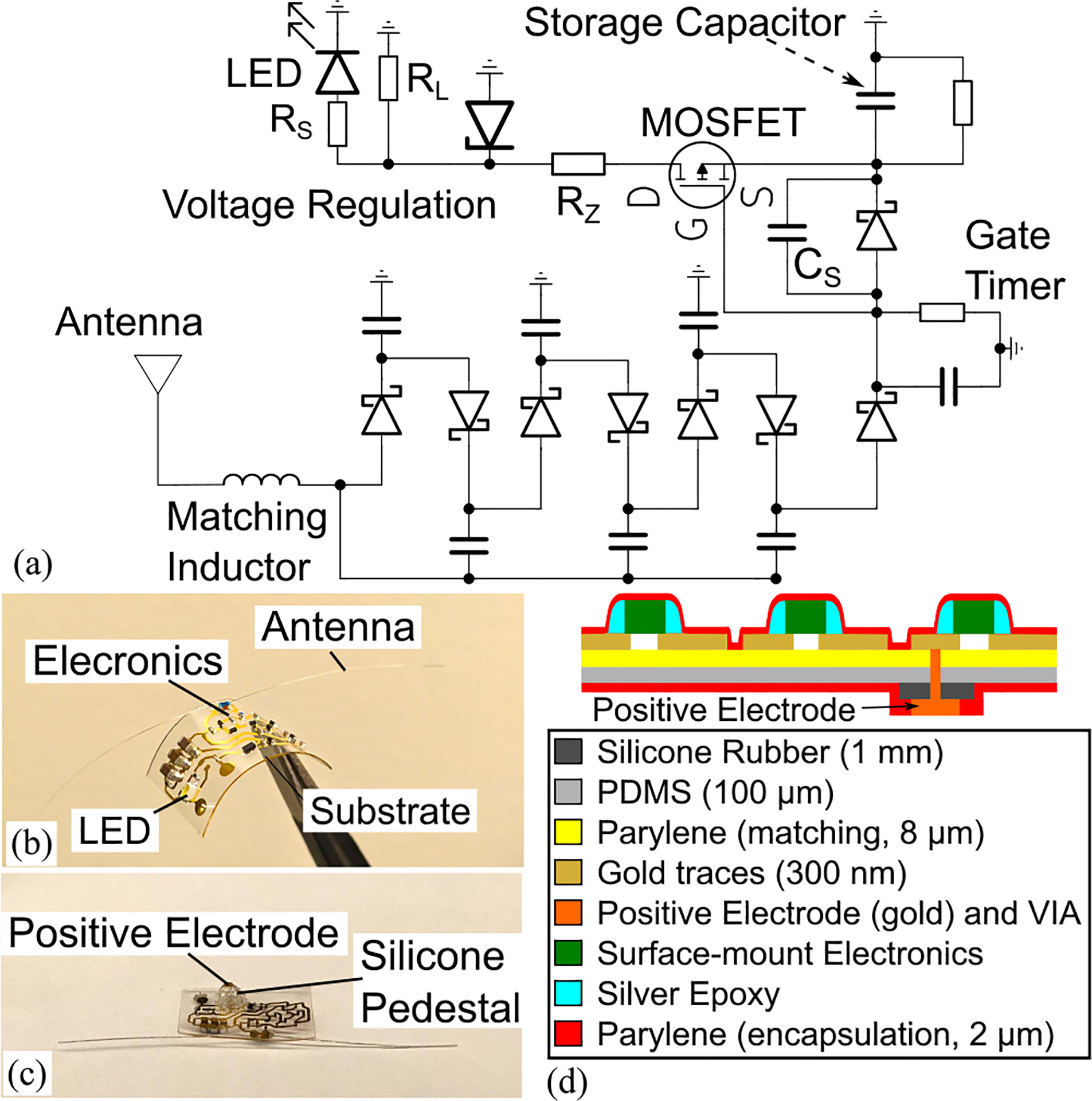
(a) Implant circuit diagram. (b) Fabricated implant top and (c) bottom view. (d) Cross-section diagram.

The implant comprises two flexible stainless steel wires making a dipole antenna (length of each wire= 24 mm and diameter= 50.8 *μ*m) operating at a center frequency of 2.4 GHz with a 30 nH RF choke inductor (20NXGRW, Coilcraft) at the feed to create a dc path to ground. The antenna was simulated in a phantom composed of the Polydimethylsiloxane (PDMS) substrate (*ϵ*_*r*_ = 2.1) located between skin (*ϵ*_*r*_ = 38), bone (*ϵ*_*r*_ = 11.4), dura (*ϵ*_*r*_ = 42.1), and gray mater (*ϵ*_*r*_ = 48.9). The simulated reflection coefficient magnitude was below −10 dB with a maximum gain of −14.5 dBi. A rectifier converts the 2.4 GHz signal into dc voltage and is shown in Fig. 2(a) Schottky diodes (JDH2S02SL, Toshiba) are assembled in a Dickson multiplier architecture with 10 pF smoothing capacitors. The rectifier circuit is interconnected using gold traces with a thickness of 300 nm and width of 400 *μ*m. An RF short capacitor (*C*_*S*_ = 20 pF) eliminates dc bias of the gate-source junction that may occur due to direct coupling of the 2.4 GHz wireless signal to the adjacent traces. The dc voltage is accumulated in storage capacitors with a value of 30 *μ*F in parallel with a 100 kΩ resistor. The gate timer circuit (*τ* = 0.1 *μ*s) controls the enhancement-type P-mosfet switch (PMZ320UPEYL, Nexperia). It discharges to enable the delivery of the stored dc voltage from the storage capacitors to the load. The load is composed of *R*_*L*_ = 10 kΩ in parallel with the LED circuit and the equivalent impedance of the stimulation current path between the positive and ground electrodes (*Z*_eq_). The stimulation current path is composed of the dura substitute with an approximate resistance of 2 kΩ when soaked in saline [18] in series with the dura and the cortex, and returns to the implant at the ground electrode. An optional voltage regulator can be implemented using a Zener diode (BZT52 C family, Diodes Incorporated) to limit the stimulation voltage amplitude. An ultra-low power LED (KW DELPS2.RA, OSRAM) operating at a wavelength of 477 nm with a series resistor (*R*_*S*_ = 804 Ω) and a forward current of 500 *μ*A enables a translational telemetry system that allows specialists to verify the implant’s operation using naked-eye observations without requiring a light detection system. In addition, it can be used to estimate the output voltage without taking wired measurements. The ground electrode is constructed using a stainless steel wire (length=10 mm and diameter = 50.8 *μ*m). It is inserted under the scalp to complete the stimulation current path.

The implant is fabricated on a PDMS substrate (184 Sylgard) (16 × 6.67 × 0.1 mm^3^), as shown in Fig. 2(b) and (c) An oxygen plasma-treated (5 min) parylene layer (Dimer-C, SCS LabCoater) is used to enhance the adhesion of the gold traces to the PDMS substrate, and the electronics are attached to the traces using conductive silver epoxy (EMS, 12642-14). The gold traces were obtained using RF sputtering (EMITECH K675X) followed by photolithography using a mylar mask (FineLine Imaging). Photolithography was performed using AZ4330 photoresist and MIF300 developer, followed by etching using GE-8148 gold etchant. A parylene encapsulation layer provides protection to the electronics and antenna while maintaining transparency to allow visual detection of the LED emission. A silicone rubber pedestal (MED-4013, NuSil) is molded using a 3D-printed resin mold (Form3, Formlabs) (thickness = 1 mm) and is attached to the bottom layer to access the dura substitute surface when the device is placed onto the outside of the skull for rodent testing, as shown in Fig. 2(d) A stainless steel via (A-M systems) (diameter = 127 *μ*m) connects the top layer to the positive electrode (Series 815–22, Mill-Max Manufacturing) at the bottom layer. The peak thickness is determined by the size of the storage capacitors which is 0.7 mm when using capacitors with a voltage tolerance of 16 V.

### B. RF Transmitter

**Fig. 3.**
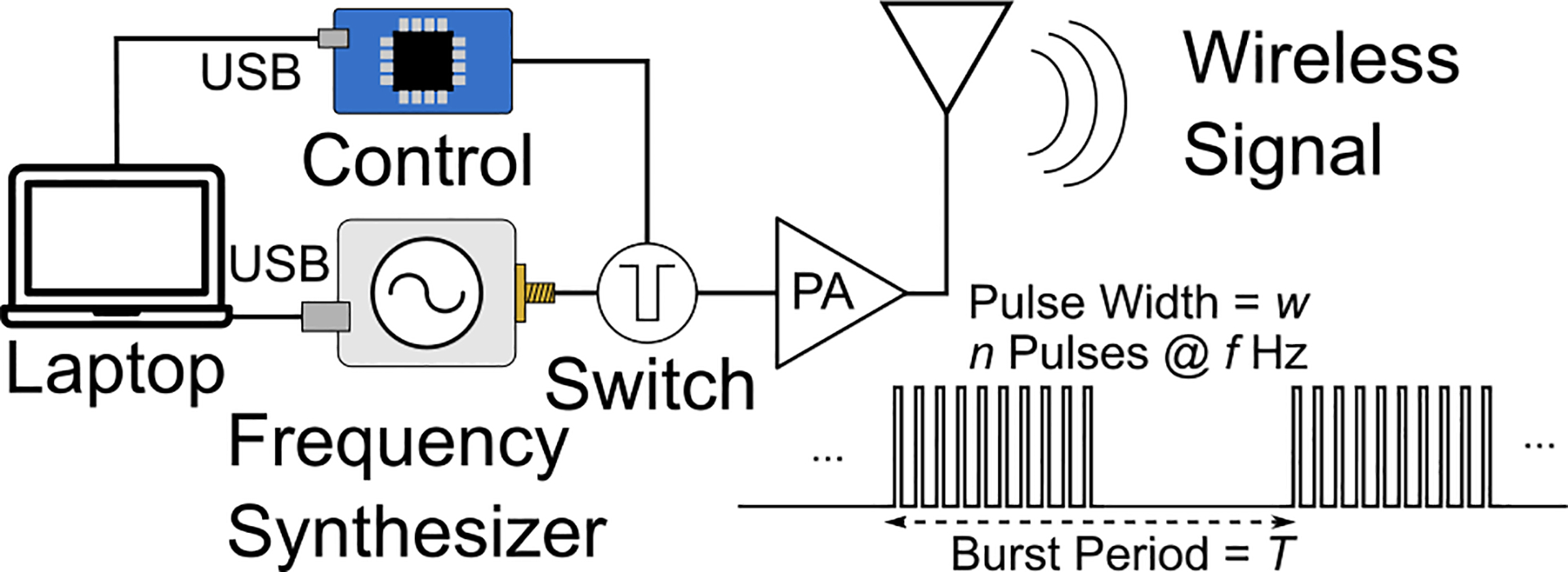
External transmitter and wireless stimulation protocol.

The RF transmitter comprises a portable frequency synthesizer (5009, Valon) that can be programmed using a laptop via a USB port. It generates a 2.4 GHz carrier that is OOK (on-off keying)-modulated using a control unit (Arduino Mega). The protocol determines the number of RF pulses (*n*) that are generated at a frequency of *f* Hz, pulse width of *w* and burst period (*T* ) as shown in Fig. 3. The implant delivers monophasic voltage pulses that resemble the transmitted stimulation protocol into the brain and the LED for optical telemetry.

### C. In Vivo Experimental Setup

**Fig. 4.**
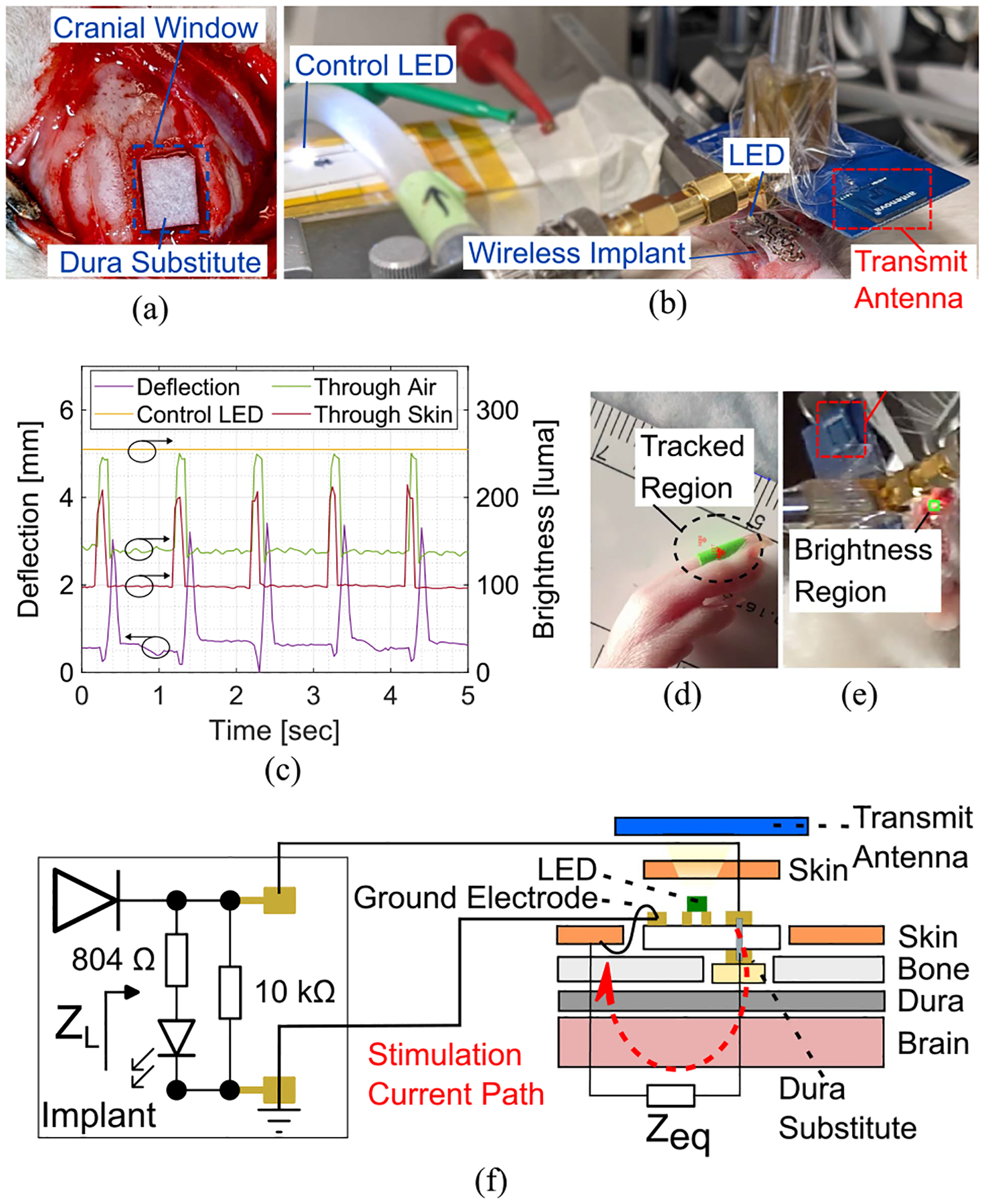
(a) Dura substitute placement. (b) Implant positioning on the dura substitute. (c) Limb deflection (left y-axis) and brightness (right y-axis) during motor cortex stimulation. (d) Limb deflection tracking. (e) Brightness detection through the skin. (f) In vivo test diagram.

The implant was tested in vivo by delivering stimulation to a rodent’s motor cortex through a dura substitute (DuraMatrix-Onlay, Stryker). A craniotomy site (3 × 7 mm^2^) was opened in the skull after administering anesthesia, and a piece of dura substitute was soaked in saline and placed in the cranial window as shown in Fig. 4(a) and (b). The implant was positioned in contact with the dura substitute and the ground electrode was located under the skin, resembling the setup in Fig. 1. A detailed experiment diagram is shown in Fig. 4(f), highlighting the load values seen by the implant and the stimulation current path. The transmit antenna (A10194, Antenova) delivers a wireless stimulation protocol with the following parameters: *n* = 10, *f* = 100 Hz, *w* = 200 *μ*s, and *T* = 1 s. In addition, a control LED is used as a brightness reference and is placed in the implant’s vicinity with *R*_*S*_ = 804 Ω and powered with 5 V dc voltage.

### D. Benchtop and In Vivo Data Acquisition

The benchtop voltage was acquired using stainless steel wires (791400, A-M Systems) connected to the positive and ground electrodes, and aligning the transmit antenna while transmitting 2.4 GHz pulses with *w* = 100 *μ*s. The voltage signals were collected using the NI DAQExpress system. The brightness was measured concurrently by analyzing video recordings in a region surrounding the LED. The test was conducted in a dark room and the setup was placed in a cardboard box to eliminate ambient light while avoiding interference with the RF signal. In the in vivo experiment, brightness was measured using the tracker open source software by analyzing video recordings to determine the brightness of a region surrounding the LED. The brightness is reported in the luma unit on a scale from 0 to 255 representing absolute black and white, respectively. A photodetector can be used as an alternative to the video camera and pixel tracker. The limb deflection was measured by marking the limb with a piece of visually distinguishable tape and tracking the movement of pixels using the tracker open source software.

## III. RESULTS

### A. Wireless Implant Benchtop Characterization

**Fig. 5.**
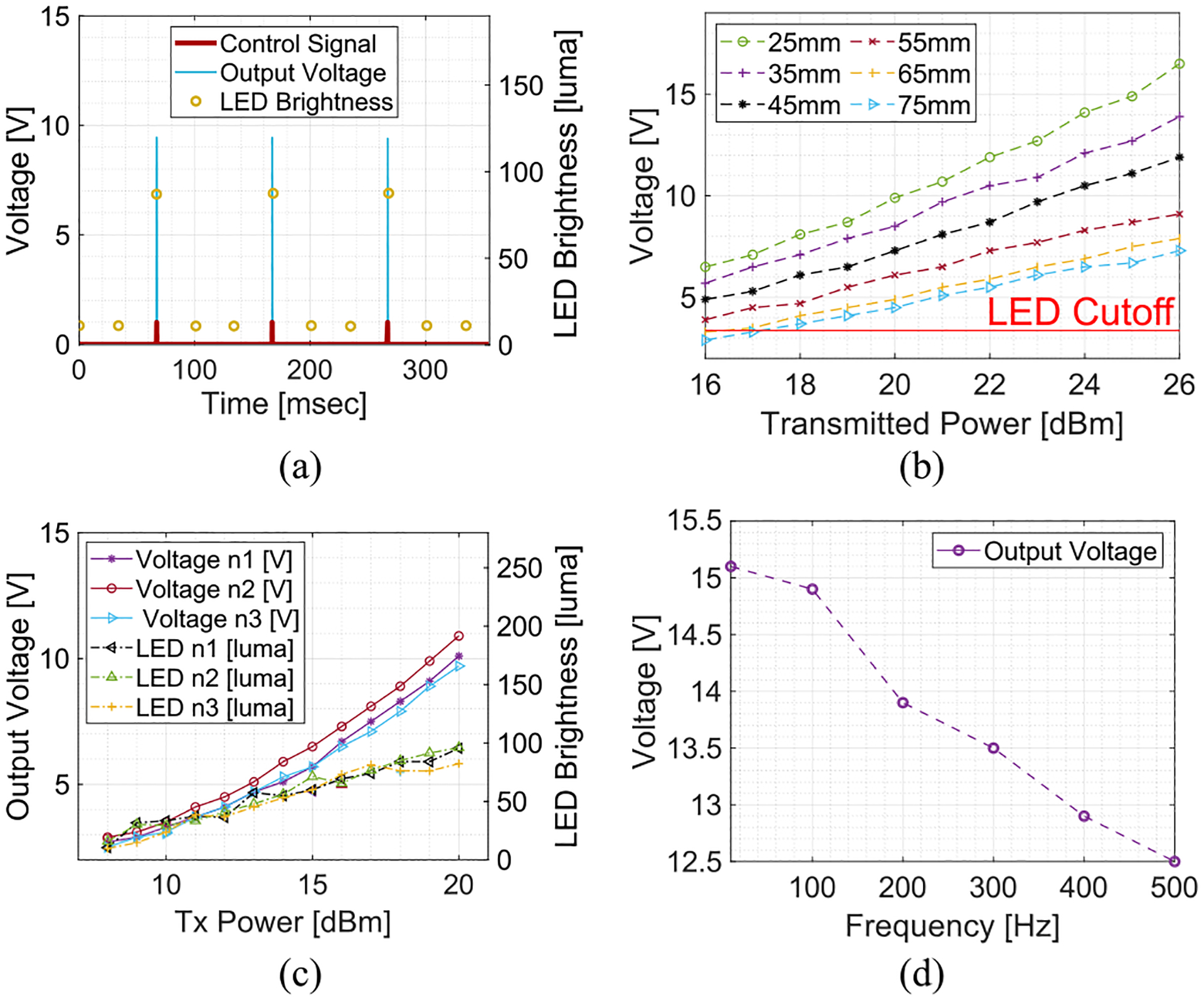
(a) Monophasic pulse generation and LED operation. (b) Output voltage as a function of power and distance. (c) Comparison of output voltage and LED brightness between three devices. (d) Output voltage as a function of burst frequency ( *f* ).

The benchtop test shows that the implant responds to the reception of the stimulation protocol by delivering monophasic voltage pulses to the load, which is composed of *R*_*L*_ = 10 kΩ in parallel with the LED circuit, as shown in Fig. 5(a). Brightness peaks of 67 luma with *R*_*S*_ = 804 Ω were distinguishable when the stimulation protocol comprised pulses with *w* = 100 *μ*s at *f* = 10 Hz. The measured pulse width is 106 *μ*s due to the delay in charging and discharging gate timer. A maximum output voltage of 16.3 V was recorded at a distance of 25 mm to the transmit antenna at a transmit power of 26 dBm. Fig. 5(b) shows the output voltage as a function of transmit power and distance to the transmit antenna. The output voltage was well above the LED cutoff voltage of 2.38 V for the majority of sweeps. The fabrication process produces consistent devices with a similar output voltage, as shown in Fig. 5(c), which exhibits the output voltage and the LED brightness from three devices when *R*_*S*_ = 15 k*Ω*. The output voltage decreases with *f* due to the insufficient time to charge the storage capacitors, as shown in Fig. 5(d). The voltage dropped by 17.2% as *f* was swept between 5 and 500 Hz. The benchtop measurements confirm that the implant can deliver adequate stimulation voltage and provide detectable brightness peaks to track the delivery of stimulation. The output voltage can be regulated when using a BZT52C10LP Zener diode (*R*_*Z*_ = 300 Ω) with a maximum voltage regulation error of 15%.

### B. In Vivo Testing Results

Wireless stimulation of the motor cortex resulted in a motor response and observable brightness peaks through air and skin mediums as shown in Fig. 4(c) with the arrows in the plot indicating the corresponding *y*-axis. Limb deflection of around 3 mm was recorded at intervals that matched the wireless stimulation protocol burst period (*T* = 1 s). Fig. 4(d) shows the tracked region that was used to record limb deflections. In addition to triggering motor response, the implant’s output voltage powers the LED to provide a visual indicator at *T* = 1 s intervals. Brightness peaks preceded limb deflections with values of 245 luma when measuring the brightness through the air medium. The measured brightness peaks in the air medium approach the control LED brightness at 254 luma, demonstrating that the wireless implant can provide adequate power through wireless power transfer even when the output voltage pulses are generated at a duty cycle of 2%. A noticeable delay of around 100 ms was observed between the brightness and deflection peaks. This delay is caused by the time required to activate descending neural pathways and trigger the limb response as shown in Fig. 4(c). The brightness region shown in Fig. 4(e) was detected when the implant was placed under skin. The measured brightness peaks decreased by 33 luma when compared with the air medium while remaining clearly visible. The brightness values measured in the in vivo setup were higher than the benchtop testing results presented in Section III–A because the test was conducted in ambient room light where the background brightness is higher. Nevertheless, the change in brightness was noticeable in the measurements through the air and skin medium. These results show that it is possible to monitor the onset of stimulation while the device is placed under the skin for future chronic studies. Before this study, we performed in vivo tests on six animals [7] using one implant with a similar structure, and we did not observe delamination or loss of consistency in output voltage.

## IV. DISCUSSION AND CONCLUSION

This letter demonstrated a wireless, battery-free implant with optical telemetry. Table 1 gives a comparison between the implant proposed in this work and similar efforts. The device features the smallest size in the discrete category, smallest substrate thickness, and peak thickness while eliminating active power. It is common to rely on a microcontroller or similar circuitry to power a telemetry unit operating in the visible [11], RF [14] or IR spectrum [15], [16]. Visible telemetry offers the advantage of naked-eye observation without the need for an external IR receiver; however, it requires good encapsulation transparency to relay the signal. Therefore, several studies explored IR and RF telemetry to eliminate the transparency constraint. The proposed implant is the only device which provides telemetry without the use of any active components that require a VDD, such as a microcontroller, multiplexer, or voltage regulator. The LED was located in parallel with the stimulation path, enabling direct visual observation of device operation. Flexible and biocompatible materials, such as polyimide, offer attractive features for wireless implants [15], [16]. The proposed implant uses PDMS as a substrate and maintains transparency. When comparing the substrate size and thickness, the proposed device features the smallest peak implant thickness of 0.7 mm owing to the elimination of large active electronics, such as a microcontroller. The substrate thickness is the smallest (equal to value reported in [15]). Integrated fabrication can further reduce the implant’s size for applications, such as rodent spinal cord testing [14]. Another distinctive feature of this work is eliminating contact between the implant and the brain. Owing to these features, the implant is an attractive option for in vivo studies that require a simple circuit architecture, small size and thickness, and telemetry for facilitated monitoring.

## Figures and Tables

**TABLE 1.** State-of-The-Art Comparison

Ref	This ‡	[11]	[14]	[16]	[15]
**Substrate Material**	Parylene/PDMS	Multiple	Multiple	Polyimide	Polyimide
**Flexible/Transparent?**	**Yes/Yes**	Yes/No	No/No	Yes/No	Yes/Yes
**Transparent Encapsulation?**	Yes	Yes	No	Yes	No
**Active? (VDD?)**	**No**	Yes	Yes	Yes	Yes
**Telemetry Type**	LED	LED	RF	IR	IR
**Tech**	Discrete	Discrete	CMOS	Discrete	Discrete
**Peak Thickness (mm)**	**0.7**	0.92	14.8	0.928	1.6
**Area (mm** ^**2**^ **)**	**106.7**	176	6.45	153.9	800
**Thickness (mm)**	**0.1**	0.17	14.8	0.128	0.1

‡ Improved metrics are bold in this column.
